# Long non-coding RNA SNHG16 affects cell proliferation and predicts a poor prognosis in patients with colorectal cancer via sponging miR-200a-3p

**DOI:** 10.1042/BSR20182498

**Published:** 2019-05-17

**Authors:** Yanling Li, Ying Lu, Yanglong Chen

**Affiliations:** 1 Department of gastroenterology, Union Hospital, Tongji Medical College, Huazhong University of Science and Technology, Wuhan 430022, Hubei Province, China; 2 Department of Emergency Surgery, Union Hospital, Tongji Medical College, Huazhong University of Science and Technology, Wuhan 430022, Hubei Province, China

**Keywords:** colorectal cancer, Long non-coding RNA, miR-200a-3p, SNHG16

## Abstract

Previous study has explored that SNHG16, a long non-coding RNA (lncRNA), mediated cell growth and proliferation. Yet, the role of SNHG16 in human colorectal cancer (CRC) still remains to be explored. Therefore, we conducted the present study to explore the functions of SNHG16 in CRC. In the present study, SNHG16 was significantly up-regulated in CRC tissues and cell lines. Gain- and loss-of-function of SNHG16 further presented that SNHG16 promoted the progression of CRC cells, including proliferation, migration, and invasion. Further, *in vivo* study also revealed that overexpression of SNHG16 could promote tumor growth. Bioinformatics analysis and luciferase reporter assay showed that SNHG16 was a direct target of miR-200a-3p. MiR-200a-3p was inversely correlated with SNHG16 expression in CRC tissues. In brief, the above results elucidate the important role of SNHG16 in CRC tumorigenesis, suggesting that SNHG16 might be quite vital for the diagnosis and development of CRC.

## Introduction

Colorectal cancer (CRC) is one of the most frequently diagnosed tumors, which ranks the third most common in the world [1]. Despite substantial advances achieved in the treatment, the overall 5 year survival rate of CRC patients still remains poor due to metastasis [2,3]. Although mounting evidence has documented that alterations in many tumor-suppressor genes and oncogenes are associated with CRC, the molecular and genetic bases of CRC remain largely unknown [4]. Therefore, identifying novel biomarkers that could predict the prognosis of CRC is urgently needed. Recently, the regulation of the non-protein-coding genome in normal physiology and the pathogenesis of diseases including cancer have attracted growing attention [5–7].

Long non-coding RNAs (lncRNAs, >200 nt in length) are generally a class of transcripts without protein-coding ability [8]. Previous studies have elucidated that lncRNAs are involved in several biological processes, such as cell proliferation, cell cycle regulation, cell differentiation, cytoplasmic transport, chromosome imprinting, and histone modification [9–12]. Increasing studies have suggested that dysfunction of lncRNAs is associated with the development and progression of cancer. For example, overexpression of lncRNA-MALAT1 was associated with poor prognosis of CRC [13]. However, an enormous number of lncRNAs remain to be elucidated and characterized.

In our study, we attempted to investigate the correlations between the expression of SNHG16 and the clinicopathological features and survival outcomes of CRC patients. Besides, the underlying molecules of SNHG16 in CRC progression were further investigated. Our results may provide new insights into understanding the molecular mechanisms of SNHG16 in CRC.

## Materials and methods

### Tissue samples

Fifty-six tumors and their matched adjacent normal tissues were selected from patients who accepted routine surgery in Union Hospital, between January 2012 and November 2016. No patients had been treated with radiotherapy or chemotherapy before surgery. All collected specimens were immediately frozen in liquid nitrogen and further stored at −80°C. Before research, we gained agreement from all participants through their assigning written informed consent. The research has been carried out in accordance with the World Medical Association Declaration of Helsinki. The study was approved by the Institutional Review Boards of the Union Hospital, Tongji Medical College.

### Cell culture

Four CRC cell lines (CaCO-2, SW480, HCT116, and LoVo cells), and the normal intestinal mucous cell line CCC-HIE-2 were obtained from the Cell Bank of Type Culture Collection (Chinese Academy of Sciences, Shanghai, China). All cells were cultured in Dulbecco’s modified Eagle’s medium (DMEM; Hyclone, Logan, UT, U.S.A.), along with 10% fetal bovine serum (Invitrogen, Grand Island, NY, U.S.A.), 100 μg/ml streptomycin, and 100 U/ml penicillin. Cells were kept at 37°C, 5% CO_2_ in an incubator.

### RNA isolation and qRT-PCR

Total RNA was extracted using Trizol reagent (Life Technologies, Carlsbad, CA, U.S.A.) using a standard procedure. Then 1 μg of RNA was reverse transcripted using PrimeScript™ RT Master Mix (TAKARA, Dalian, China), according to the manufacturer’s instructions. Real-time PCR was employed to determine the relative expression level of target genes by the SYBR-Premix ExTaq II kit (Takara) on 7900HT fast Real-time PCR system (Applied Biosystems, Foster City, CA, U.S.A.). The relative expression level of lncRNA, and miRNA were normalized against GAPDH and U6 small nuclear RNA. The primers were SNHG16, F: CAGAATGCCATGGTTTCCCC, R: TGGCAAGAGACTTCCTGAGG; snRNA U6 F: GCTTCGGCAGCACATATACTAAAAT, R: CGCTTCACGAATTTGCGTGTCAT; GAPDH F: GGGCTGCTTTTAACTCTGGTAAAG, R: CCATGGGTGGAATCATATTGG. Each assay was performed in triplicate independent experiments.

### Transfection

All plasmid vectors for transfection were extracted by DNA Midiprep kit (Qiagen, Hilden, Germany). Three individual SNHG16 siRNAs (si-SNHG16) and scrambled negative control siRNA (si-NC) were purchased from Ribo BioCoLTD (Guangzhou, China). The target sequences of siRNA were as follows: si-NC, 5′-UUCUCCGAACGUGUCACGUTT-3′; si-SNHG16 1, 5′-GGAAUGAAGCAACUGAGAUUU-3′; si-SNHG16 2, 5′-CATGTCCTTCTGATCACCAAGTTGACTTA-3′; si-SNHG16 3, 5′-GATATCTTAGTCCTAACCATATTGATCCC-3′. Lipofectamine™ 2000 transfection reagent (Invitrogen, U.S.A.) was used to transfected oligonucleotide and plasmid, followed by the manufacturer’s protocol. After transfection for 48 h, cells were further used in the relevant experiments. Plasmid complementary DNA SNHG16 cDNA (pcDNA-SNHG16) was constructed by amplification and introduction of SNHG16 cDNA sequence into the pcDNA3.1 vector (Invitrogen).

### Cell proliferation assay

The proliferation assay was evaluated using the MTT assay according to the manufacturer’s instructions. After transfection with either pcDNA-SNHG16, pcDNA empty vector, siRNA SNHG16 or siRNA NC, LoVo and SW480 cells were seeded in 96-well plates at a density of 5000 cells/well. After cultured for 24–96 h, the transfected cells proliferation was treated with 30 μl/well of MTT solutions. After an additional incubation for 4 h, the resulting formazan was dissolved in 100 ml of DMSO. The spectrophotometric absorbance was measured at 570 nm. Each assay was repeated three times.

### Colony formation assays

Colony formation assay was performed as previously described [14]. Briefly, after transfection for 24 h, cells were seeded into six-well plates at a density of 500 cells per well. After consecutively cultured for 14 days at 37°C, the cells were fixed with methanol and then stained with 0.2% crystal violet. Visible formed colonies with >50 cells were counted by light microscopy.

### Migration and invasion assays

The migration and invasion abilities of cells were assessed using the transwell chamber (8 μm pore size, Millipore). 2 × 10^4^ cells in serum-free medium were seeded into the upper chamber after transfection. The lower chamber was supplied with medium containing 10% FBS. After incubation for 24 h, cells in the upper chamber were wiped out, followed by fixation with 4% formaldehyde for staining with 0.1% crystal violet. As invasion assay, the upper chamber was pre-coated with 30 µl of Matrigel (BD Biosciences, Heidelberg, Germany) with the other experimental procedures the same as the migration assay. The numbers of membrane-penetrating cells were snapped in three randomly fields using an IX71 inverted microscope (Olympus, Tokyo, Japan).

### Western blot analysis

After treatment, total proteins were extracted from cells using RIPA lysis buffer containing protease inhibitors. Protein concentrations were measured by Bradford dye (Bio-Rad). 40 μg proteins were loading to the SDS-PAGE gel, and then transferred to PVDF membranes (Millipore, Billerica, MA, U.S.A.). Then, the PVDF membranes were blocked by 5% milk and further immersed in primary antibodies overnight at 4°C. After washing with TBST, the membranes were incubated with the secondary antibody (Cell Signaling, U.S.A.) (1:5000 dilution) for 1 h at RT. After TBST wash, ECL solutions (Thermo Pierce) were used to detect the protein bands. GAPDH was served as an endogenous control. Antibodies used include E-cadherin (ab1416, 1:2000 dilution), Vimentin (ab92547, 1:2000 dilution), β-catenin (ab32572, 1:2000 dilution), and GAPDH (ab8245, 1:3000 dilution) were purchased from Abcam (U.S.A.). α-SMA was purchased from Cell Signaling (Cell Signaling 19245, 1:2000 dilution).

### Dual luciferase reporter assay

Two reporter plasmids were constructed by inserting SNHG16 fragments with wild-type (WT) or mutant (MUT) binding sites of miR-200a-3p into the pmiR-RB-REPORT™ (Ribobio, Guangzhou, China). Cells (2.0 × 10^4^) cultured in 96-well plates were co-transfected with 200 ng miR-200a-3p or empty vector, 50 ng firefly luciferase reporter containing SNHG16 wild-type or SNHG16 mutant, and 2 ng of pRL-TK (Promega, Madison, WI, U.S.A.) using Transfection reagent Lipofectamine 2000 (Invitrogen) according to the protocol. Luciferase activity was assayed 48 h after transfection, using a dual luciferase reporter assay system (Promega).

### RNA immunoprecipitation

RNA immunoprecipitation (RIP) was performed using thermo fisher RIP kit (Thermo, U.S.A.) based on the manufacturer’s protocol. The Ago2 antibodies are purchased from Abcam (U.S.A.). Purified RNA was subjected to qRT-PCR analysis to demonstrate the presence of the binding targets using respective primers.

### Animal work

Six male BALB/c nude mice aged 6 weeks were purchased from Slac Laboratory Animal Center (Shanghai, China) and maintained under specific pathogen free (SPF) condition in the animal care facility. A total of 3 × 10^6^ LoVo cells transfected with pcDNA/SNHG16 or control were suspended in 0.2 ml Matrigel Matrix (BD Biosciences) and then subcutaneously injected into the flanks of two group mice (*n*=3). Tumor diameter was recorded using a vernier caliper every 3 days. Tumor volume was calculated follows formula: tumor volume (mm^3^) = (length × width^2^)/2. At day 24, mice were killed and tumor weights were recorded.

### Statistical analysis

starBase was used to predict the target of SNHG16. The significance of differences between two groups was estimated using Student’s *t*-test and the *χ*^2^-test. Survival analysis was assessed using Kaplan–Meier method. The correlations between SNHG16 expression and clinicopathologic factors were evaluated using Chi-Square test. *P*<0.05 was considered as statistically significant. The software GraphPad Prism 6 was used for statistical analyses.

## Results

### SNHG16 expression is up-regulated in CRC tissues and associated with disease progression

**Figure 1:**
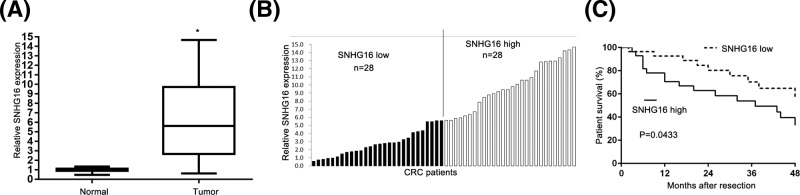
Expression of SNHG16 in CRC tissues and cell lines (**A**) The expression of SNHG16 in 56 CRC tissues and adjacent noncancerous tissues was analyzed by qPCR and normalized to GAPDH expression *P<0.05. (**B**) Fifty-six CRC patients were divided into SNHG16 high group (*n*=28) and SNHG16 low group (*n*=28) according to the median expression level. (**C**) The relationship between levels of SNHG16 and overall survival was analyzed by Kaplan–Meier survival analysis.

The expression level of SNHG16 in 56 pairs of CRC tissues and adjacent non-tumor tissues was explored using qRT-PCR. Figure 1A revealed that SNHG16 level was significantly higher in CRC tissues compared with normal tissues. We further divided patients into two groups: high expression group (*n*=28) and the low expression group (*n*=28), based on the median value of SNHG16 (Figure 1B). The relationship of SNHG16 with various clinical features of CRC was analyzed and is summarized in Table 1. It could be informed that the expression of SNHG16 was related to metastasis (*P*=0.0482), and lymph node (*P*=0.0002). However, we failed to observe any relationship between SNHG16 and other clinicopathological characteristics (gender, age, smoking, and tumor range). We further performed Kaplan–Meier analyses to further explore the correlation of SNHG16 expression with overall survival of CRC patients. The results showed that overall survival time of high SNHG16 expression group was significantly shorter than that of low SNHG16 expression group (*P*<0.05, Figure 1C).

**Table 1:** Correlation between lncRNA-SNHG16 expression and clinical features (*n*=56)

Factor	Relative SNHG16 expression level		*P* value
	High (*n*=28)	Low (*n*=28)	
Gender			0.0801
Males	20	19	
Females	8	9	
Age, year			0.5920
≤60	16	14	
>60	12	14	
Smoking			0.1789
No smoking	10	15	
Smoking	18	13	
Tumor range			0.1812
T1–T3	11	16	
≥T4	17	12	
Metastasis			0.0482
Negative	13	6	
Positive	15	22	
Lymph nodes			0.0002*
Negative	6	20	
Positive	22	8	

*P<0.05.

### SNHG16 promotes cell proliferation of CRC cells

**Figure 2:**
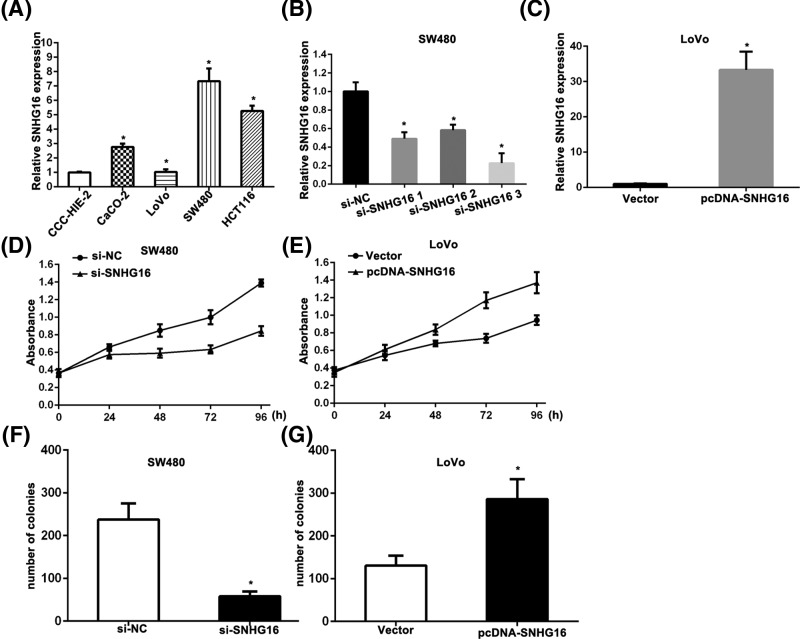
Effect of SNHG16 on cell proliferation in CRC cells (**A**) qRT-PCR was performed to detect the relative expression of SNHG16 in normal cell CCC-HIE-2 and CRC cells CaCO-2, LoVo, SW480, and HCT116. (**B**) The knockdown efficiency of SNHG16 specific siRNA was determined by qRT-PCR. (**C**) The over-expression of SNHG16 was determined by qRT-PCR after transfection with pcDNA-SNHG16. (**D,E**) MTT assay was performed to detect the proliferation ability of CRC cells after transfected with si-SNHG16 and pcDNA-SNHG16. (**F,G**) Colony formation assay was performed to detect the proliferation ability of CRC cells after transfected with si-SNHG16 and pcDNA-SNHG16. ^*^P<0.05 versus controls.

Next, we determined the relative expressions of SNHG16 among four CRC cell lines (SW480, LoVo, CaCO-2, and HCT116 cells), as well as the normal intestinal mucous cell line CCC-HIE-2. The results showed that SW480 expressed the highest levels of SNHG16 and LoVo expressed the lowest levels of SNHG16, compared with the other cell lines, respectively (Figure 2A). To silence SNHG16 expression in SW480 cells, three siRNAs were synthesized and transfected into SW480. siRNA 3 could decrease most, and thus was selected in the further experiments (Figure 2B). After transfection for 48 h, qRT-PCR analysis revealed that SNHG16 expression was up-regulated in LoVo cells, compared with control cells (Figure 2C). We further performed MTT assay to evaluate the potential effects of SNHG16 on cell proliferation. MTT assay showed that down-regulation of SNHG16 could inhibit cell growth of SW480 cells, while up-regulation of SNHG16 promoted LoVo cells growth, compared with respective controls (Figure 2D,E). Similarly, colony formation assay results revealed that the colony numbers of SW480 cells transfected with si-SNHG16 and LoVo cells transfected with pcDNA/SNHG16 were significantly lower and higher than those transfected with respective controls (Figure 2F,G).

### SNHG16 promotes migration and invasion of CRC cells

**Figure 3:**
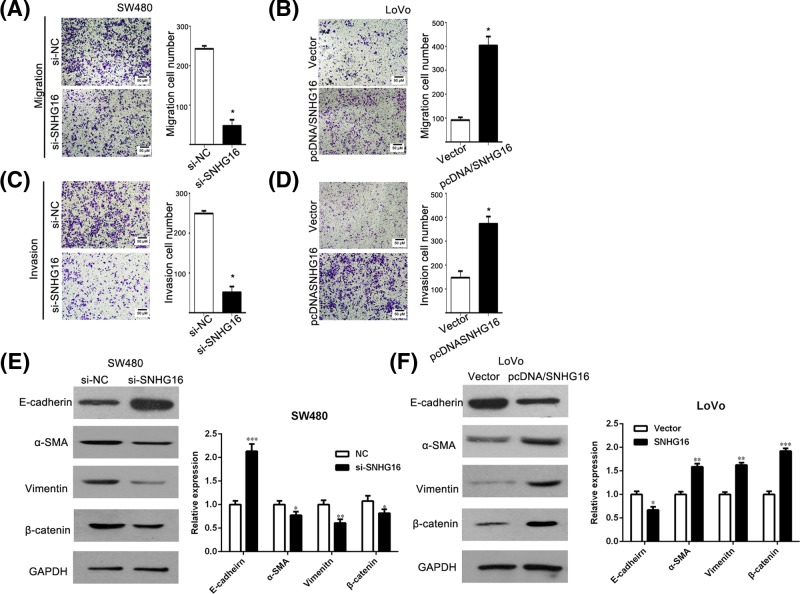
Effects of SNHG16 on migration and invasion in CRC cells (**A,B**) Detection for cell migration ability of CRC cells after transfected with si-SNHG16 and pcDNA-SNHG16. (**C,D**) Detection for invasion ability of CRC cells after transfected with si-SNHG16 and pcDNA-SNHG16. ^*^P<0.05. (**E,F**) The expression level of EMT-related gene (E-cadherin, α-SMA, Vimentin, and β-catenin) was detected by Western blot.**P<0.05, ***P<0.05.

We further performed the cell migration and invasion assay to determine the malignant characteristics of SNHG16. The migration assays showed that the migratory ability of SW480 cells was inhibited as indicated by the decrease in migrated cells, after transfected by si-SNHG16 (Figure 3A). A similar result also was observed in the invasion assay (Figure 3C). To the contrary, LoVo cells transfected with pcDNA/SNHG16 vector migrated more (Figure 3B). Similar results were also obtained in invasion assay (Figure 3D). The results demonstrated that SNHG16 promotes migration and invasion of CRC cell. Therefore, to further evaluate whether SNHG16 can promote epithelial to mesenchymal transition (EMT) processes in CRC cancer, we examined the expression of EMT markers α-SMA, vimentin, E-cadherin, and β-catenin expression by Western blotting. The data suggested that SNHG16 up-regulated mesenchymal markers α-SMA and vimentin expression and promoted the nuclear translocation of β-catenin, while decreased the expression of epithelial markers E-cadherin, indicating that it promotes EMT processes (Figure 3E,F).

### SNHG16 promotes tumor growth *in vivo*

**Figure 4:**
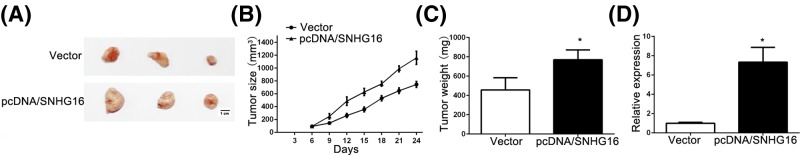
Effect of SNHG16 on tumor growth *in vivo* (**A**) The tumor morphology was present. (**B**) Tumor size was measured every 3 days. (**C**) Tumor weight was detected. ^*^P<0.05. (**D**) The relative expression of SNHG16 was detected in the tumors. ^*^P<0.05.

To examine the potential role of SNHG16 in tumorigenicity, we observed the growth process of a CRC cancer-cell xenograft. Injection of LoVo cells with stably overexpressed SNHG16 resulted in a dramatic growth in tumor size when compared with controls (Figure 4A–C). The expression of SNHG16 in tumor after transfected with pcDNA/SNHG16 was higher than the controls (Figure 4D).

### SNHG16 is a target of miR-200a-3p

**Figure 5:**
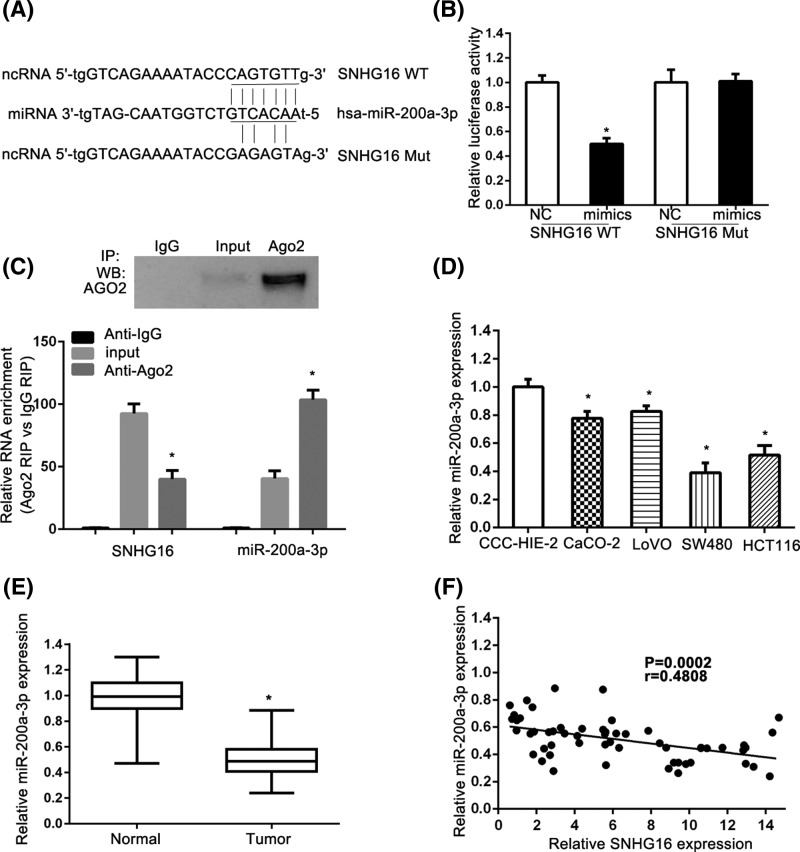
Identification of SNHG16 as a target of miR-200a-3p (**A**) The miR-200a-3p binding site predicted in the 3′UTR of SNHG16. (**B**) Relative Firefly/Renilla luminescence mediated by luciferase plasmid harboring the wild-type or mutant SNHG16 sequence upon transfection with miR-200a-3p expression plasmid. Data are means ± SD of three independent experiments. ^*^P<0.05. (**C**) Cells lysates were collected for RIP using Ago2 antibody. IP-Western blot and qRT-PCR were used to detect Ago2, SNHG16, and miR-200a-3p. (**D**) Relative expression of miR-200a-3p in CRC cell lines compared with normal cell line. The relative level of miR-200a-3p was normalized against U6 small nuclear RNA. Data are means ± SD of three independent experiments. (**E**) The level of miR-200a-3p in CRC tissues was measured. (**F**) The expression correlation between miR-200a-3p and SNHG16 in CRC tissues was analyzed by Spearman’s correlation analysis (*R*^2^ = 0.2312, *P*=0.0002). ^*^P<0.05.

Accumulating documents have been demonstrated that many lncRNAs are identified as ceRNAs for specific miRNAs. We used the online software starBase [15,16] to predict and select miRNAs interacted with SNHG16. SNHG16 was predicted as a target of miR-200a-3p using starBase (Figure 5A). We performed the luciferase reporter assay to confirm such interaction. As shown in Figure 5B, CRC cells transfected with miR-200a-3p mimics and reporter plasmids containing the wide type SNHG16 significantly suppressed the luciferase activity when compared with cells transfected with scrambled miRNA and reporter plasmids containing the wild type SNHG16 in LoVo cells. Co-transfection with miR-200a-3p mimics and mutated SNHG16 had no effect on the luciferase activity when compare to cells co-transfected with scrambled miRNA and mutated SNHG16. miRNAs are present in the cytoplasm in the form of miRNA ribonucleoprotein complexes (miRNPs) containing Ago2 protein, the core component of the RNA-induced silencing complex (RISC). Moreover, lncRNAs can function as a molecular sponge or a ceRNA to regulate miRNA level by interacting with RISC. The RNA binding protein immunoprecipitation (RIP) experiments on LoVo cell extracts were further performed. The Ago2 antibody precipitated the Ago2 protein was first confirmed from our cellular extract (Figure 5C, upper panel). Moreover, we found that SNHG16 and miR-200a-3p were enriched significantly, in Ago2 pellets relative to control IgG immunoprecipitates (Figure 5C, lower panel). The level of miR-200a-3p was lower in the CRC cell lines, compared with the normal cell line (Figure 5D). The PCR results indicated that the level of miR-200a-3p was lower in the cancer tissues when compared with that in adjacent normal tissues (Figure 5E). Finally, the Spearman’s correlation analysis showed that the level of miR-200a-3p was negatively correlated with the level of SNHG16 (Figure 5F).

## Discussion

As CRC is still prevailing in China, it remains an urgent clinical challenge to elucidate molecular mechanism underlying CRC carcinogenesis. In recent years, lncRNAs have attracted a lot of research interest from all over the world [17,18]. Growing bodies of evidence suggest that some lncRNAs are associated with cancer development [19,20]. Yet, the role of lncRNA in cancer are different, some of them might be tumor suppressor, some might be tumor promoter. Several lncRNAs have been found in playing critical role in CRC [21–23]. Therefore, further studies in identifying more novel lncRNAs in CRC is of great need.

Previous studies have proved that lncRNA SNHG16 was increased in ovarian cancer tissues and cell lines, and high SNHG16 expression in ovarian cancer was associated with poorer prognosis [24]. Cai et al. demonstrated that lncRNA SNHG16 was frequently higher in breast cancer tissues than in the paired noncancerous tissues. Moreover, they found that SNHG16 significantly promoted breast cancer cell migration by competitively binding miR-98 with E2F5 [25]. However, the value of SNHG16 in clinical practice of CRC is unknown.

In the present study, we attempted to explore the functional role of SNHG16 in CRC. We first investigated the relevant expression level of SNHG16 using the clinical samples. The clinical results showed that the expression of SNHG16 in CRC tissues was significantly up-regulated, when compared with adjacent normal tissues. We further explored the relationships between SNHG16 expression levels and clinicopathological characteristics. We found that SNHG16 expression was significantly associated with remarkably associated with metastasis (*P*=0.0482), and lymph node (*P*=0.0002). Moreover, we found that SNHG16 might act as a prognostic factor for survival in CRC patients. However, we found that low expression of SNHG16 associates with patients being positive for metastasis. Such phenomenon might be due to the relative small sample size. Further explorations still need to be performed.

To further elucidate the underlying mechanism of SNHG16 in CRC, we applied loss-of-function and gain-of-function approaches. Down-regulation of SNHG16 could suppress cell proliferation, colony formation, migration and invasion in CRC cell lines *in vitro*. However, overexpression of SNHG16 promoted the growth, migration, and invasion of CRC. It is well known that aberrant cadherins and vimentin expression was involved in metastasis. As expected, the results showed that SNHG16 could promote cell migration and invasion with up-regulation of α-SMA, β-catenin, and vimentin. Previous study revealed that miR-200a *in vitro* led to a significantly differential and converse expression of EMT-related genes [26], whether SNHG16 functions through miR-200a-3p waits to reveal.

Inspired by the ‘competitive endogenous RNAs’ regulatory network and the growing proof about lncRNAs’ role in regulatory circuitry [27–29], we proposed that SNHG16 might act as a ceRNA to regulate relevant cellular control. Therefore, we adopted bioinformatics analysis and luciferase assays to verify the potential target of SNHG16. Interestingly, we found that miR-200a-3p could form complementary base pairing with SNHG16 and induce translational repression of a SNHG16 reporter gene. Moreover, SNHG16: miR-200a-3p coimmunoprecipitation with anti-Ago2 demonstrated a direct approval of interacting with each other. A study performed by Christensen et al. revealed that SNHG16 is significantly up-regulated in adenomas and all stages of CRC [30]. Their results demonstrate that SNHG16 may play an oncogenic role in CRC and that it affects genes involved in lipid metabolism, possible through ceRNA related mechanisms. Their conclusions were quite similar to ours, yet we further explore the *in vivo* role of SNHG16.

## Conclusions

In all, here we first investigated the role of SNHG16 in CRC carcinogenesis. Our study revealed that SNHG16 could promote CRC cell growth and invasion. We found that SNHG16 was associated with the malignancy and poor prognosis in CRC. We also indicated miR-200a-3p was the target of SNHG16. These results may undoubtedly further help to elucidate the etiology of CRC initiation and progression.

## Abbreviations

CRC: colorectal cancer
EMT: epithelial to mesenchymal transition
IncRNA: long non-coding RNA
RIP: RNA immunoprecipitation
RISC: RNA-induced silencing complex

## References

[1] Torre L.A., Bray F., Siegel R.L., Ferlay J., Lortet-Tieulent J. and Jemal A. (2015) Global cancer statistics, 2012. CA Cancer J. Clin. 65, 87–108 10.3322/caac.21262 25651787

[2] Roncucci L. and Mariani F. (2015) Prevention of colorectal cancer: how many tools do we have in our basket? Eur. J. Intern. Med. 26, 752–756 10.1016/j.ejim.2015.08.019 26499755

[3] Brody H. (2015) Colorectal cancer. Nature 521, S1 10.1038/521S1a 25970450

[4] Obuch J.C. and Ahnen D.J. (2016) Colorectal cancer: genetics is changing everything. Gastroenterol. Clin. North Am. 45, 459–476 10.1016/j.gtc.2016.04.005 27546843

[5] Martens-Uzunova E.S., Bottcher R., Croce C.M., Jenster G., Visakorpi T. and Calin G.A. (2014) Long noncoding RNA in prostate, bladder, and kidney cancer. Eur. Urol. 65, 1140–1151 10.1016/j.eururo.2013.12.003 24373479

[6] Arase M., Horiguchi K., Ehata S., Morikawa M., Tsutsumi S., Aburatani H. (2014) Transforming growth factor-beta-induced lncRNA-Smad7 inhibits apoptosis of mouse breast cancer JygMC(A) cells. Cancer Sci. 105, 974–982 10.1111/cas.12454 24863656 PMC4317863

[7] An X., Sarmiento C., Tan T. and Zhu H. (2017) Regulation of multidrug resistance by microRNAs in anti-cancer therapy. Acta Pharm. Sin. B 7, 38–51 10.1016/j.apsb.2016.09.002 28119807 PMC5237711

[8] Wu Z., Liu X., Liu L., Deng H., Zhang J., Xu Q. (2014) Regulation of lncRNA expression. Cell Mol. Biol. Lett. 19, 561–575 10.2478/s11658-014-0212-6 25311814 PMC6275606

[9] Ghazal S., McKinnon B., Zhou J., Mueller M., Men Y., Yang L. (2015) H19 lncRNA alters stromal cell growth via IGF signaling in the endometrium of women with endometriosis. EMBO Mol. Med. 7, 996–1003 10.15252/emmm.201505245 26089099 PMC4551339

[10] Liu J.Y., Yao J., Li X.M., Song Y.C., Wang X.Q., Li Y.J. (2014) Pathogenic role of lncRNA-MALAT1 in endothelial cell dysfunction in diabetes mellitus. Cell Death Dis. 5, e1506 10.1038/cddis.2014.466 25356875 PMC4649539

[11] Rodriguez-Malave N.I., Fernando T.R., Patel P.C., Contreras J.R., Palanichamy J.K., Tran T.M. (2015) BALR-6 regulates cell growth and cell survival in B-lymphoblastic leukemia. Mol. Cancer 14, 214 10.1186/s12943-015-0485-z 26694754 PMC4688921

[12] Yang L., Lin C., Jin C., Yang J.C., Tanasa B., Li W. (2013) lncRNA-dependent mechanisms of androgen-receptor-regulated gene activation programs. Nature 500, 598–602 10.1038/nature12451 23945587 PMC4034386

[13] Zheng H.T., Shi D.B., Wang Y.W., Li X.X., Xu Y., Tripathi P. (2014) High expression of lncRNA MALAT1 suggests a biomarker of poor prognosis in colorectal cancer. Int. J. Clin. Exp. Pathol. 7, 3174–3181 25031737 PMC4097248

[14] Fahrioglu U., Dodurga Y., Elmas L. and Secme M. (2016) Ferulic acid decreases cell viability and colony formation while inhibiting migration of MIA PaCa-2 human pancreatic cancer cells in vitro. Gene 576, 476–482 10.1016/j.gene.2015.10.061 26516023

[15] Yang J.H., Li J.H., Shao P., Zhou H., Chen Y.Q. and Qu L.H. (2011) starBase: a database for exploring microRNA-mRNA interaction maps from Argonaute CLIP-Seq and Degradome-Seq data. Nucleic Acids Res. 39, D202–D209 10.1093/nar/gkq1056 21037263 PMC3013664

[16] Li J.H., Liu S., Zhou H., Qu L.H. and Yang J.H. (2014) starBase v2.0: decoding miRNA-ceRNA, miRNA-ncRNA and protein-RNA interaction networks from large-scale CLIP-Seq data. Nucleic Acids Res. 42, D92–D97 10.1093/nar/gkt1248 24297251 PMC3964941

[17] Han D., Wang M., Ma N., Xu Y., Jiang Y. and Gao X. (2015) Long noncoding RNAs: novel players in colorectal cancer. Cancer Lett. 361, 13–21 10.1016/j.canlet.2015.03.002 25754818

[18] Huarte M. (2015) The emerging role of lncRNAs in cancer. Nat. Med. 21, 1253–1261 10.1038/nm.3981 26540387

[19] Shi S.J., Wang L.J., Yu B., Li Y.H., Jin Y. and Bai X.Z. (2015) LncRNA-ATB promotes trastuzumab resistance and invasion-metastasis cascade in breast cancer. Oncotarget 6, 11652–11663 10.18632/oncotarget.3457 25871474 PMC4484483

[20] Wang P., Ning S., Zhang Y., Li R., Ye J., Zhao Z. (2015) Identification of lncRNA-associated competing triplets reveals global patterns and prognostic markers for cancer. Nucleic Acids Res. 43, 3478–3489 10.1093/nar/gkv233 25800746 PMC4402541

[21] Iguchi T., Uchi R., Nambara S., Saito T., Komatsu H., Hirata H. (2015) A long noncoding RNA, lncRNA-ATB, is involved in the progression and prognosis of colorectal cancer. Anticancer Res. 35, 1385–1388 25750289

[22] Liao Q., He W., Liu J., Cen Y., Luo L., Yu C. (2015) Identification and functional annotation of lncRNA genes with hypermethylation in colorectal cancer. Gene 572, 259–265 10.1016/j.gene.2015.07.028 26172871

[23] Shi J., Li X., Zhang F., Zhang C., Guan Q., Cao X. (2015) Circulating lncRNAs associated with occurrence of colorectal cancer progression. Am. J. Cancer Res. 5, 2258–2265 26328256 PMC4548337

[24] Yang X.S., Wang G.X. and Luo L. (2018) Long non-coding RNA SNHG16 promotes cell growth and metastasis in ovarian cancer. Eur. Rev. Med. Pharmacol. Sci. 22, 616–622 29461589 10.26355/eurrev_201802_14284

[25] Cai C., Huo Q., Wang X., Chen B. and Yang Q. (2017) SNHG16 contributes to breast cancer cell migration by competitively binding miR-98 with E2F5. Biochem. Biophys. Res. Commun. 485, 272–278 10.1016/j.bbrc.2017.02.094 28232182

[26] Pichler M., Ress A.L., Winter E., Stiegelbauer V., Karbiener M., Schwarzenbacher D. (2014) MiR-200a regulates epithelial to mesenchymal transition-related gene expression and determines prognosis in colorectal cancer patients. Br. J. Cancer 110, 1614–1621 10.1038/bjc.2014.51 24504363 PMC3960623

[27] Broderick J.A. and Zamore P.D. (2014) Competitive endogenous RNAs cannot alter microRNA function in vivo. Mol. Cell 54, 711–713 10.1016/j.molcel.2014.05.023 24905003

[28] Guo G., Kang Q., Zhu X., Chen Q., Wang X., Chen Y. (2015) A long noncoding RNA critically regulates Bcr-Abl-mediated cellular transformation by acting as a competitive endogenous RNA. Oncogene 34, 1768–1779 10.1038/onc.2014.131 24837367

[29] Karreth F.A., Reschke M., Ruocco A., Ng C., Chapuy B., Leopold V. (2015) The BRAF pseudogene functions as a competitive endogenous RNA and induces lymphoma in vivo. Cell 161, 319–332 10.1016/j.cell.2015.02.043 25843629 PMC6922011

[30] Christensen L.L., True K., Hamilton M.P., Nielsen M.M., Damas N.D., Damgaard C.K. (2016) SNHG16 is regulated by the Wnt pathway in colorectal cancer and affects genes involved in lipid metabolism. Mol. Oncol. 10, 1266–1282 10.1016/j.molonc.2016.06.003 27396952 PMC5423192

